# Accumulation of Renal Fibrosis in Hyperuricemia Rats Is Attributed to the Recruitment of Mast Cells, Activation of the TGF-β1/Smad2/3 Pathway, and Aggravation of Oxidative Stress

**DOI:** 10.3390/ijms241310839

**Published:** 2023-06-29

**Authors:** Mingkang Zhang, Ruirui Cui, Yan Zhou, Yanrong Ma, Yongwen Jin, Lina Wang, Wen Kou, Xin’an Wu

**Affiliations:** 1 School of Pharmacy, Lanzhou University, Lanzhou 730000, China; 2 Engineering Research Centre of Prevention and Control for Clinical Medication Risk, Lanzhou 730000, China; 3 Department of Pharmacy, The First Hospital of Lanzhou University, Lanzhou 730000, China; 4 Department of Radiotherapy, The First Hospital of Lanzhou University, Lanzhou 730000, China

**Keywords:** hyperuricemia, renal fibrosis, mast cells, TGF-β1/Smad2/3 pathway, oxidative stress

## Abstract

Renal fibrosis is relentlessly progressive and irreversible, and a life-threatening risk. With the continuous intake of a high-purine diet, hyperuricemia has become a health risk factor in addition to hyperglycemia, hypertension, and hyperlipidemia. Hyperuricemia is also an independent risk factor for renal interstitial fibrosis. Numerous studies have reported that increased mast cells (MCs) are closely associated with kidney injury induced by different triggering factors. This study investigated the effect of MCs on renal injury in rats caused by hyperuricemia and the relationship between MCs and renal fibrosis. Our results reveal that hyperuricemia contributes to renal injury, with a significant increase in renal MCs, leading to renal fibrosis, mitochondrial structural disorders, and oxidative stress damage. The administration of the MCs membrane stabilizer, sodium cromoglycate (SCG), decreased the expression of SCF/c-kit, reduced the expression of α-SMA, MMP2, and inhibited the TGF-β1/Smad2/3 pathway, thereby alleviating renal fibrosis. Additionally, SCG reduced renal oxidative stress and mitigated mitochondrial structural damage by inhibiting Ang II production and increasing renal GSH, GSH-Px, and GR levels. Collectively, the recruitment of MCs, activation of the TGF-β1/Smad2/3 pathway, and Ang II production drive renal oxidative stress, ultimately promoting the progression of renal fibrosis in hyperuricemic rats.

## 1. Introduction

Uric acid (UA) is the end product of purine metabolism in humans. Excessive accumulation of UA leads to hyperuricemia and the deposition of urate crystals in various tissues, particularly the kidneys. Hyperuricemia is an independent risk indicator for kidney disease [1] and contributes to the development of chronic kidney disease (CKD) [2]. Approximately 70% of the UA produced daily is excreted by the kidneys, and hyperuricemia may be generated when the production and excretion of UA are not in equilibrium [3]. According to the 2007–2008 NHANES survey, the prevalence of hyperuricemia among US adults is 21%. However, its prevalence is increasing rapidly, considering lifestyle and dietary factors (obesity, alcohol, fructose, and fatty foods rich in purines) [4]. Similarly, the overall prevalence of hyperuricemia in Chinese adults was reported to be 12.69%, and the number was significantly higher in men compared to women [5]. Apparently, hyperuricemia has become a life-threatening element along with hyperglycemia, hyperlipidemia, and hypertension [6]. More and more researchers are focusing on the occurrence and progression of hyperuricemia.

Nephropathy caused by hyperuricemia is characterized by renal calculi, interstitial nephritis, and fibrosis [7]. Fibrosis represents a dysfunctional wound-healing process or excessive scarring, mainly characterized by the progressive accumulation of fibrous tissue and even loss of organ function [8]. Fibrosis affects nearly every organ, including the kidneys, liver, heart, and lungs, and accounts for 45% of all deaths in the industrialized world. Moreover, the fibrosis process is considered relentlessly progressive and irreversible [9]. Hyperuricemia is also an independent risk factor for interstitial fibrosis [10]. It is likely related to its induction of oxidative stress and activation of the renin–angiotensin system (RAS) [11]. Angiotensin II (Ang II), a systemic regulator of RAS, directly contributes to the development of renal disease [12] and has emerged as an essential pro-fibrotic cytokine in renal fibrosis [13].

Furthermore, renal fibrosis is a chronic and progressive pathological process, and there is not yet a targeted therapy to mitigate renal fibrosis [14]. Transforming growth factor-β1 (TGF-β1) plays a vital role in renal fibrosis. It facilitates the production of renal extracellular matrix (ECM), and the excessive deposition of ECM in the renal interstitium ultimately leads to renal fibrosis [15]. It has been demonstrated that multiple interactions exist between TGF-β and Ang II. For instance, directly and indirectly, Ang II increases the transcription and synthesis of TGF-β, further promoting the formation of EMT [16]. Smad2/3 signaling is closely associated with renal fibrosis. It can encourage the fibrotic response by binding to specific promoter regions of collagen genes and either directly promoting ECM production or inhibiting ECM degradation. Concurrently, activation of TGF-β1 triggers nuclear localization of Smad2/3 in tubular epithelial cells and fibroblasts and triggers pro-fibrotic gene overexpression [17]. An overwhelming amount of research has shown that the TGF-β1/Smad2/3 pathway is an important pathogenic mechanism for tissue fibrosis [18].

MCs are located in the skin, on the mucosal surface of the intestine, and in other vascularized organs (e.g., the liver and kidney) [19]. MCs are essential immune cells that participate in innate and adaptive immunity. Accumulating experimental evidence has suggested that MCs contribute to cardiac fibrosis, possibly due to the secretion of specific proteases (chymase and tryptase), promoting the production of Ang II, or producing TGF-β by direct or indirect pathways [20]. Additional studies have also reported that MCs are responsible for renal fibrosis, including diabetic kidney disease (DKD) [21], obesity-induced kidney damage [22], hypertension-induced renal impairment [23], unilateral ureteral obstruction (UUO) [24], and adefovir accumulation [25]. Moreover, MC-deficient mice had lower levels of collagen deposition in the kidney caused by UUO, and decreased expression of pro-fibrogenic factors was also observed [26]. Unfortunately, although MCs play a non-negligible role in kidney injury, few studies have investigated the association between MCs and fibrosis due to hyperuricemia, and the mechanisms involved are poorly understood.

In this study, we established a kidney injury model of hyperuricemia rats with a continuous high-purine diet for three weeks. Then, we administered corresponding different doses of sodium cromolyn (SCG) or valsartan (VAL) for another three weeks. We aimed to explore the relationship between MCs and hyperuricemia-induced renal fibrosis. The association of MCs and the TGF-β1/Smad2/3 pathway was also investigated. This study may provide new ideas and approaches for treating hyperuricemia-induced renal injury. 

## 2. Results

### 2.1. Effect of Administration of Uric Acid on the Release of Ang II from RBL-2H3 Cells

**Figure 1:**
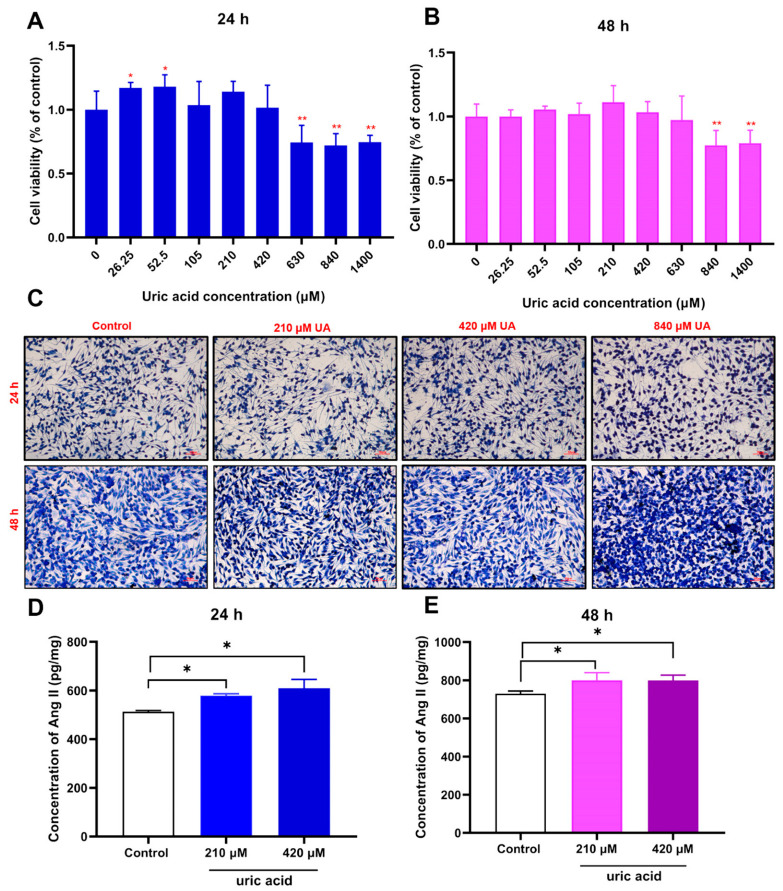
Effect of administration of uric acid on the release of Ang II from RBL-2H3 cells. (**A**,**B**): The viability of RBL-2H3 cells was determined via CCK-8 assay after treatment with various uric acid concentrations for 24 h or 48 h. (**C**): Toluidine blue staining of RBL-2H3 after treatment with various uric acid concentrations for 24 h or 48 h. Scale bar = 50 µm. (**D**,**E**): Ang II levels in each group after treatment with various uric acid concentrations for 24 h or 48 h. Results are shown as mean ± S.D. (*n* = 4). * *p* < 0.05, ** *p* < 0.01 indicate statistically significant differences compared with the control group.

Mast cells (RBL-2H3 cells) are commonly employed for in vitro studies [27,28]. To investigate whether uric acid directly affects the release of Ang II from RBL-2H3 cells, we conducted in vitro experiments of uric acid stimulation of RBL-2H3 cells. As shown in Figure 1A,B, the cell viability results showed that incubating RBL-2H3 cells with 210 or 420 μM uric acid for 24 or 48 h had no apparent effect on the viability of these cells. Moreover, toluidine blue staining did not reveal any change in the morphology of RBL-2H3 cells after incubation with uric acid, as described above (Figure 1C). We then selected the above uric acid concentrations to culture RBL-2H3 cells for 24 or 48 h, respectively, and assayed the release of Ang II. The Elisa assay results showed that treatment with either 210 or 420 μM uric acid for 24 or 48 h could promote the release of Ang II from RBL-2H3 cells (*p* < 0.05, Figure 1D,E). These results indicate that high uric acid concentrations promote the production of Ang II from RBL-2H3 cells.

### 2.2. Effects of SCG or VAL on Renal Function in Hyperuricemic Rats

**Figure 2:**
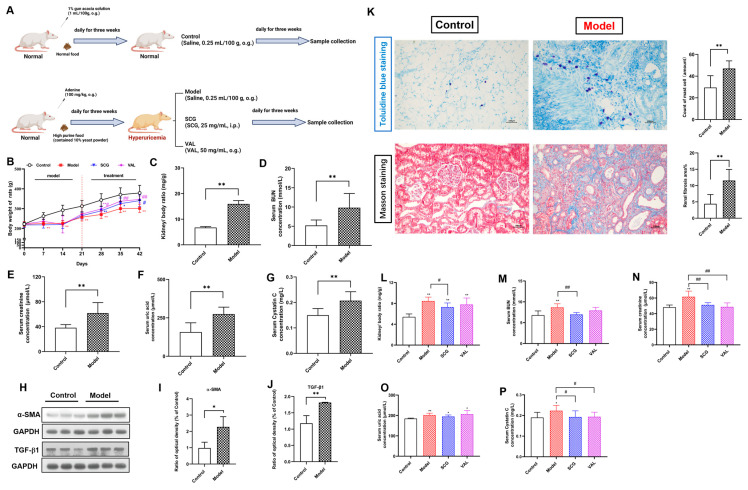
Effects of SCG or VAL on biochemical parameters of rats in each group. (**A**): Protocols and time scales for animal experiments. (**B**): Body weight of rats in each group. (**C**–**G**): The ratio of the kidney to body weight of rats, the concentrations of serum BUN, creatinine, uric acid, and cystatin C in rats with a model of hyperuricemia after three weeks. (**H**–**J**): The expression of renal TGF-β1and α-SMA in each group. (**K**): Toluidine blue staining and Masson staining of renal slices in each group (200×). (**L**–**P**): The ratio of the kidney to body weight of rats in each group, the concentrations of serum BUN, creatinine, uric acid, and cystatin C in each group. Results are shown as mean ± S.D. (*n* = 7). * *p* < 0.05, ** *p* < 0.01 indicate statistically significant differences compared with the control group. # *p* < 0.05, ## *p* < 0.01 indicate statistically significant differences compared with the model group.

In vivo, experiments were subsequently carried out in SD rats (Figure 2A). First, all animals were subjected to a high purine diet for three weeks to construct a rat model of hyperuricemia. As shown in Figure 2B, the body weight of the rats decreased significantly after three weeks of renal injury induced by the high purine diet compared to the normal rats (*p* < 0.01). Notably, there was a significant increase in body weight after three weeks of coadministration with SCG or VAL compared to untreated rats (*p* < 0.05). In addition, the ratio of the kidney to body weight, serum BUN, creatinine, uric acid, and cystatin C levels in the model group were significantly increased after three weeks of modeling hyperuricemia (*p* < 0.01, Figure 2C–G). The expression of both renal TGF-β1 and α-SMA was also detected to be significantly increased (*p* < 0.05, Figure 2H–J), which demonstrated that a model of renal fibrosis injury induced by hyperuricemia had been successfully established. After that, we observed renal MCs recruitment and fibrosis by toluidine blue staining and Masson staining, respectively. The results are shown in Figure 2K. We found that compared with the control group, the recruitment and number of MCs in the kidneys of hyperuricemia rats were significantly increased (*p* < 0.01), and severe fibrotic damage occurred in the kidneys. In stark contrast to the above, after three weeks of coadministration with SCG or VAL, serum creatinine, and cystatin C levels were significantly decreased (*p* < 0.05). Moreover, significant reductions in the ratio of the kidney to body and serum BUN levels were also observed after three weeks of SCG administration (*p* < 0.05, Figure 2L–P). These results suggested that MCs were closely related to hyperuricemia-induced renal injury, and the coadministration of SCG or VAL may alleviate the progression of hyperuricemia-induced renal injury.

### 2.3. Effects of SCG or VAL on Renal Histopathology in Hyperuricemic Rats

**Figure 3:**
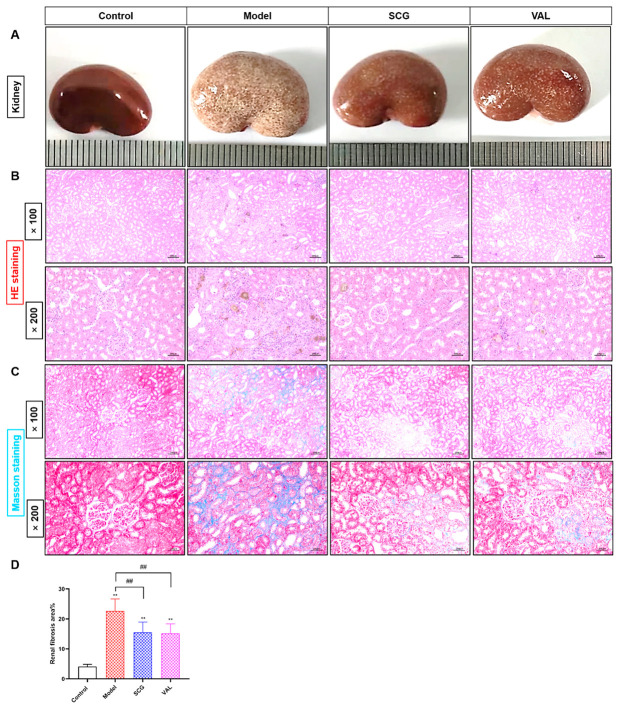
Effects of SCG or VAL on renal pathological changes in hyperuricemic rats. (**A**): Appearance of the kidneys of rats in each group. (**B**): HE staining of rat kidneys in each group (×200). (**C**): Masson staining of rat kidneys in each group (×200). Scale bar = 100 µm. (**D**): Renal fibrosis area was measured using Image J software (National Institutes of Health, Bethesda, MD, USA). Results are shown as mean ± S.D. (*n* = 7). ** *p* < 0.01 indicate statistically significant differences compared with the control group. ## *p* < 0.01 indicate statistically significant differences compared with the model group.

In response to changes in renal function indicators, we observed histopathological changes in the kidney. Notably, the renal appearance of hyperuricemic rats changed visibly after the coadministration of SCG or VAL (Figure 3A). HE and Masson staining were also used to investigate the histopathological changes in the kidney. HE staining results were shown in Figure 3B. Compared with the control group, extensive urate crystal accumulation, tubular dilatation and vacuolar degeneration, and inflammatory cell infiltration were observed in the kidneys of the model group. The above pathological damage was significantly improved after the coadministration of SCG or VAL. In addition, Masson staining was used to evaluate renal fibrosis, as shown in Figure 3C,D, which showed a large amount of fibrosis deposition in the kidney of the model group. However, renal fibrosis was reduced after the coadministration of SCG or VAL (*p* < 0.01). The results of renal histopathology were concordant with those of biochemical analysis. These results have strongly indicated that the coadministration of SCG or VAL could effectively alleviate the progression of renal injury associated with hyperuricemia.

### 2.4. MCs Recruitment in the Kidney of Hyperuricemic Rats

**Figure 4:**
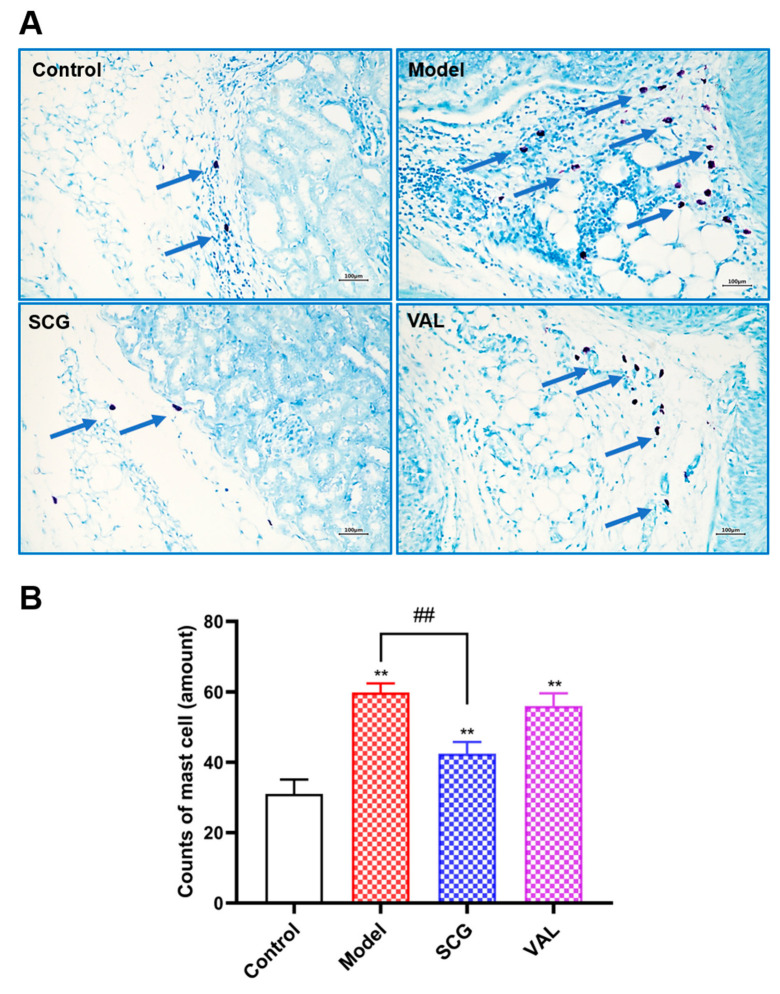
Effects of SCG or VAL on the number of renal MCs in hyperuricemic rats. (**A**): Toluidine blue staining of MCs of renal slices in each group (200×). The blue arrows represent MCs. (**B**): Number of MCs in toluidine blue staining of kidney sections. Results are shown as mean ± S.D. (*n* = 7). ** *p* < 0.01 indicate statistically significant differences compared with the control group. ## *p* < 0.01 indicate statistically significant differences compared with the model group.

Toluidine blue staining is performed for specific staining of MCs [29]. Since MCs are closely linked to kidney damage, we investigated the number and distribution of MCs in the kidney with the support of toluidine blue staining. As shown in Figure 4, compared with the control group, there was a statistically significant increase in the number of renal MCs (*p* < 0.01). In contrast, the number of MCs decreased considerably after SCG administration (*p* < 0.01). In combination with the measurements of biochemical functions, our study demonstrated a correlation between MCs and hyperuricemia-induced kidney injury.

### 2.5. Effects of SCG or VAL on the Expression of Renal SCF/C-Kit in Hyperuricemic Rats

**Figure 5:**
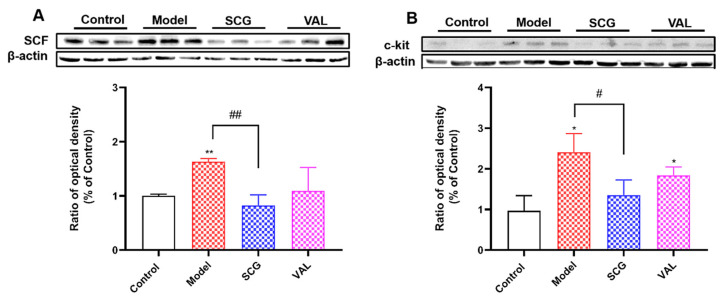
Effects of SCG or VAL on the expression of renal SCF/c-kit in hyperuricemic rats. (**A**): The expression of renal SCF in each group. (**B**): The expression of renal c-kit in each group. Results are shown as mean ± S.D. (*n* = 3). * *p* < 0.05, ** *p* < 0.01 indicate statistically significant differences compared with the control group. # *p* < 0.05, ## *p* < 0.01 indicate statistically significant differences compared with the model group.

C-kit receptors are localized on the plasma membrane of MCs and are receptors for SCF. Activation of the c-kit is critical for MCs’ growth, differentiation, and migration [30]. Given the significant increase in the number of renal MCs in hyperuricemic rats, we measured the expression of renal SCF/c-kit. The results showed that compared with the control group, the expression of SCF/c-kit in the kidney of rats in the model group was significantly increased (*p* < 0.05, Figure 5). However, after the coadministration of SCG, the expression of SCF and c-kit significantly decreased (*p* < 0.01). The c-kit ligand SCF is mainly expressed in renal collecting duct cells (https://www.proteinatlas.org/ENSG00000049130-KITLG/single+cell+type/kidney: accessed on 1 June 2022), whereas c-kit is expressed on MCs. The increased c-kit level in the kidneys of rats in the model group corresponded to MCs recruitment shown previously by toluidine staining. The effects of SCG and VAL on SCF are interesting and might be of mechanistic interest in conditioning the kidney duct cells. These results suggest that the coadministration of SCG inhibited the recruitment of MCs in the kidney of hyperuricemic rats by suppressing the expression of SCF/c-kit. 

### 2.6. Effects of SCG or VAL on Renal Fibrosis in Hyperuricemic Rats

**Figure 6:**
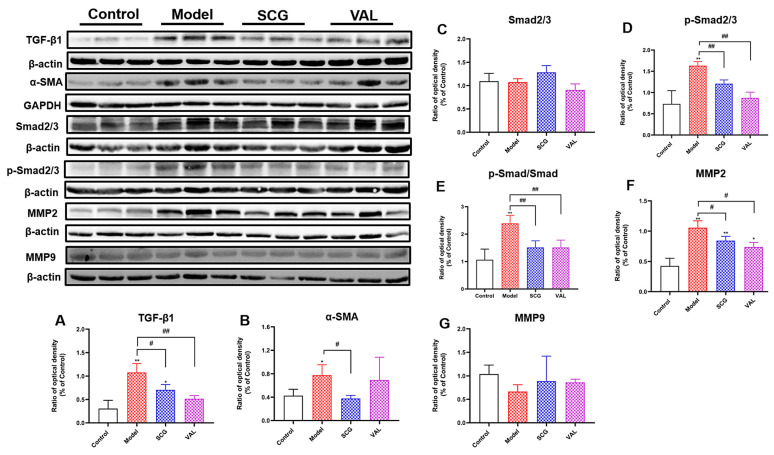
Effects of SCG or VAL on the expression of renal fibrosis factors in hyperuricemic rats. (**A**–**G**): The expression of renal TGF-β1, α-SMA, Smad2/3, p-Smad2/3, p-Smad/Smad, MMP2, and MMP9 in each group. Results are shown as mean ± S.D. (*n* = 3). * *p* < 0.05, ** *p* < 0.01 indicate statistically significant differences compared with the control group. # *p* < 0.05, ## *p* < 0.01 indicate statistically significant differences compared with the model group.

TGF-β1/Smasd2/3 pathway plays an essential part in the development of renal fibrosis [31,32]. To further explore the effect of SCG or VAL on renal fibrosis in hyperuricemic rats, we measured the expressions of fibrosis factors TGF-β1 and α-SMA. The results are shown in Figure 6A,B and showed that the expression of TGF-β1 and α-SMA in the model group was significantly increased (*p* < 0.05). Compared with the model group, the expression of TGF-β1 and α-SMA was decreased considerably after SCG administration (*p* < 0.05), and the expression level of TGF-β1 was also significantly reduced after VAL administration (*p* < 0.01). We further measured the expression of Smad2/3 and p-Smad2/3, and the results showed that compared with the control group, the expression of Smad2/3 in the model group, SCG, and VAL groups were not significantly different. In contrast, the expression of p-Smad2/3 in the model group was significantly increased (*p* < 0.01). Compared with the model group, after SCG or VAL administration, the expression of p-Smad2/3 was significantly decreased (*p* < 0.01, Figure 6C,D). Then, we compared the changes of p-Smad/Smad. As a result, we found that compared with the control group, the p-Smad/Smad level in the model group was significantly increased (*p* < 0.01). After the administration of SCG or VAL, the significantly increased p-Smad/Smad level was significantly reduced (*p* < 0.01, Figure 6E). In addition, the expression of MMP2/9 in the kidney was also examined. The results showed that the expression of MMP2 was significantly increased in the model group (*p* < 0.01), and the expression of MMP9 was not significantly changed. In contrast, the expression of MMP2 was significantly decreased after the coadministration of SCG or VAL (*p* < 0.05, Figure 6F,G). The results show that the coadministration of SCG or VAL inhibited renal fibrosis in hyperuricemic rats by suppressing the TGF-β1/Smad2/3 pathway and decreasing MMP2 production to reduce ECM formation.

### 2.7. Effects of SCG or VAL on Mitochondria of Renal Tubular Epithelial Cells in Hyperuricemic Rats

**Figure 7:**
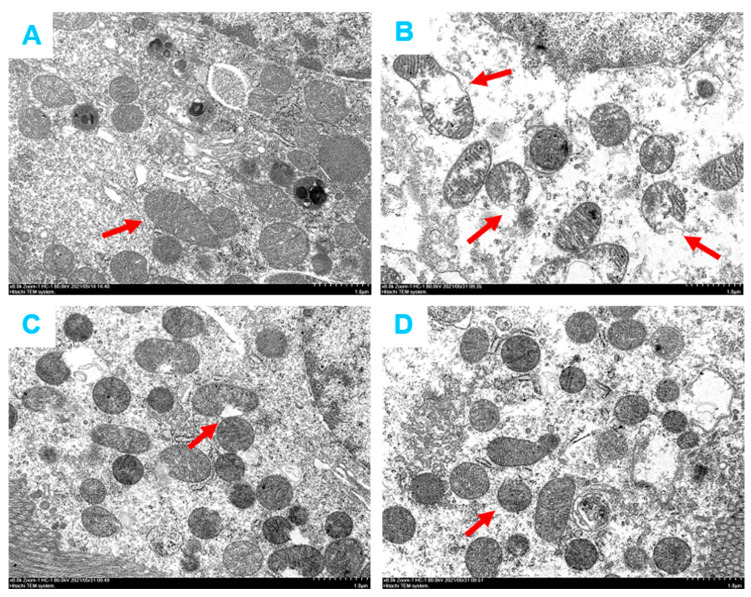
The microstructure of renal tubular epithelial cells in hyperuricemic rats. (**A**): Control group. (**B**): Model group. (**C**): SCG group. (**D**): VAL group. Scale bar = 5 µm.

The kidneys are second only to the heart regarding mitochondrial counts and oxygen consumption [33]. Since mitochondria play a non-negligible role in energy supply, kidney disease is also considered a mitochondria-related pathology [34], we used transmission electron microscopy (TEM) to observe the structure of mitochondria in renal tubular epithelial cells. As shown in Figure 7, compared with the control group, fragmented mitochondria with irregular and disordered shapes and vacuoles (red arrow) were observed in the kidney of the model group. After the coadministration of SCG or VAL, the mitochondrial structural damage of the above renal tubular epithelial cells was significantly improved. The results indicated that the coadministration of SCG or VAL attenuated hyperuricemia-induced renal mitochondrial damage. 

### 2.8. Effects of SCG or VAL on Renal Oxidative Stress in Hyperuricemic Rats

**Figure 8:**
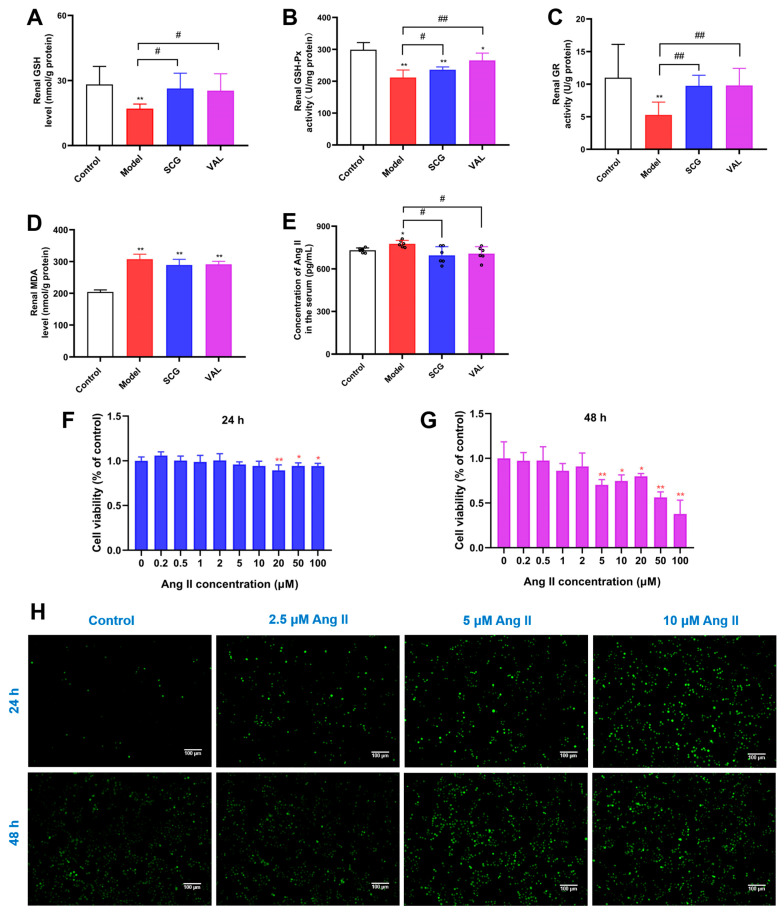
Effects of SCG or VAL on renal oxidative stress in hyperuricemic rats. (**A**–**D**): Renal GSH level, renal GSH-Px activity, renal GR activity, and renal MDA level in each group. (**E**): Serum Ang II level in each group. (**F**,**G**): The viability of HK-2 cells was determined via CCK-8 assay after treatment with various Ang II concentrations for 24 h or 48 h. (**H**): Reactive oxygen species (ROS) levels in each group after incubation of HK-2 cells with different concentrations of Ang II for 24 or 48 h (100×). Results are shown as mean ± S.D. (*n* = 6). * *p* < 0.05, ** *p* < 0.01 indicate statistically significant differences compared with the control group. # *p* < 0.05, ## *p* < 0.01 indicate statistically significant differences compared with the model group.

Oxidative stress is defined as the dysregulation of antioxidant mechanisms and the overproduction of reactive oxygen species (ROS) [35]. The kidney is a mitochondria-rich organ, and mitochondria are the primary site of aerobic respiration of cells, so the kidney is vulnerable to oxidative stress injury [36]. There are two types of antioxidant systems in the body to protect biological systems from the toxic effects of free radicals. One is a non-enzymatic antioxidant system consisting mainly of vitamins C, E, and glutathione (GSH). The other is the enzymatic antioxidant system, which mainly includes superoxide dismutase (SOD) and glutathione peroxidase (GSH-Px) [37]. Lipid peroxides, including malondialdehyde (MDA), are formed when the body resists oxidative stress through enzymatic or non-enzymatic systems. The level of MDA indirectly reflects the severity of the oxidative attack on the body [38]. Levels of SOD, GSH-Px, and glutathione reductase (GR) are often considered indicators of the body’s antioxidant capacity, mainly because SOD converts superoxide into hydrogen peroxide [39]. GSH-Px promotes the reaction of hydrogen peroxide with reduced glutathione to form H_2_O and oxidized glutathione (GSSG), which is then converted back to GSH by GR [40]. As the alterations of renal GSH, GSH-Px, GR, and MDA levels are often used to assess the extent of oxidative stress injury, we detected these levels in the kidneys of each group. The results are shown in Figure 8A–D. We found that the renal GSH, GSH-Px, and GR levels were significantly decreased (*p* < 0.01), and MDA levels were significantly increased (*p* < 0.01) in the model rats compared with the control group. In contrast to the above, GSH, GSH-Px, and GR levels were significantly increased after the coadministration of SCG or VAL (*p* < 0.05). Ang II is thought to cause renal oxidative stress through structural and functional changes in the peritubular microvasculature [16]. Thus, the changes in the Ang II level were also subsequently detected by Elisa kit (Figure 8E). We found that serum Ang II levels were significantly elevated in the model group (*p* < 0.01), while the elevated Ang II levels decreased significantly with the coadministration of SCG or VAL (*p* < 0.05). 

In addition, we designed different concentrations of Ang II to incubate HK-2 cell lines for 24 or 48 h, and cell viability was assayed using the CCK-8 assay. HK-2 cells were then incubated with Ang II (2.5, 5 or 10 μM) for 24 or 48 h. The levels of reactive oxygen species (ROS) were detected by fluorescence microscopy. We found that the effect of Ang II on the survival rate of HK-2 cells was time- and dose-dependent (Figure 8F,G). Moreover, high concentrations of Ang II directly promoted the production of ROS (Figure 8H). These results revealed that the coadministration of SCG or VAL alleviated the renal oxidative stress damage caused by hyperuricemia by inhibiting the production of Ang II.

## 3. Discussion

Hyperuricemia can lead to severe kidney damage, including obstructive nephropathy, glomerulosclerosis, and interstitial fibrosis. The primary mechanisms involved include fibrosis, endothelial dysfunction, kidney inflammation, and oxidative stress damage [1]. Hyperuricemia is a common complication of CKD, and it is also associated with CKD’s development and progression, and hyperuricemia’s prevalence negatively correlates with renal function [41]. Our results found that increased BUN, creatinine, and cystatin C levels were significantly reduced after combined SCG administration. Similarly, the elevated creatinine and cystatin C levels also decreased significantly after administering VAL. These results suggested that the combination of SCGS or VAL may alleviate the progression of renal injury caused by hyperuricemia.

Although clinically indicated UA reduction strategies can prevent or slow the progression of CKD, unwanted effects, such as severe skin adverse reactions associated with allopurinol, often make the therapeutic outcome less than satisfactory [42]. MCs are derived from hematopoietic stem cells, but MCs that have just entered the peripheral blood circulation from the bone marrow are in an immature state. Notably, immature MCs migrate through the blood to target tissues, where they mature in response to tissue-specific factors [43]. A large number of studies have demonstrated that MCs are closely associated with diabetic nephropathy [44], IgA nephropathy [45], hypertensive nephropathy [46], and obesity-related nephropathy [22]. The present study also investigated the relationship between MCs and renal injury caused by hyperuricemia. We first found a statistically significant increase in the number of MCs in hyperuricemic rats in the results of toluidine blue staining of kidney sections. After the hyperuricemia model was successfully established for three weeks, we administered SCG daily for another three weeks. It was found that the coadministration of SCG significantly reduced the recruitment of MCs compared with the model group. These results indicated that coadministration of SCG attenuated hyperuricemia-induced renal injury by stabilizing MCs.

SCF is also known as stem cell growth factor (SCGF) or mast cell growth factor (MGF). SCF is a receptor for the proto-oncogene c-kit (c-kit) tyrosine kinase receptor and is expressed on the surface of MCs [47]. SCF regulates the proliferation, differentiation, survival, and activation of MCs. Activating the c-kit leads to the accumulation of MCs in tissues [19]. SCF and its receptor c-kit are closely associated with tissue inflammation, remodeling, and fibrosis progression [48]. Moreover, SCF is an important chemokine that drives the recruitment of MCs or their precursors from the bone marrow to the sites of tumor, fibrosis, and immune inflammation [47]. Yin et al. have found that MCs infiltration promotes interstitial fibrosis in diabetic nephropathy through the SCF/c-kit pathway and that inhibition of the SCF/c-kit pathway may alleviate interstitial fibrosis [21]. A recent study by Qi et al. found that a youthful blood environment downregulated SCF/c-kit expression in aged UUO mice, improved renal fibrosis, and restored impaired renal function [49]. As with their results, our study found that the expression of SCF/c-kit was significantly elevated in the kidneys of hyperuricemic rats. The coadministration of SCG significantly reduced the expression of elevated SCF/c-kit levels. Furthermore, the results of HE staining of renal pathology similarly demonstrated that SCG could alleviate the progression of renal injury caused by hyperuricemia. These results indicated that MCs were involved in hyperuricemia-induced renal injury and that coadministration of SCG could stabilize MCs and reduce their recruitment by decreasing the expression of SCF/c-kit, thereby alleviating renal injury. 

Renal fibrosis is a pathological feature of CKD, and hyperuricemia is an important independent risk factor for its induction [50]. The most striking pathological features of kidney damage due to hyperuricemia are uric acid kidney stones, chronic interstitial nephritis, and fibrosis [7]. In our study, the results of Masson staining in renal pathology demonstrated that persistent hyperuricemia caused severe renal fibrosis and that the coadministration of SCG or VAL significantly alleviated the fibrotic damage. TGF-β1 and MMP2/9 are essential factors in renal fibrosis, which promotes excessive deposition of extracellular matrix (ECM) and promotes renal fibrosis [15]. Numerous studies have reported that TGF-β1 has a critical role in promoting the pathogenicity of fibrosis through Smad-dependent signaling [51]. The expression of pro-fibrotic TGF-β1, α-SMA, and MMP2/9 was also examined to evaluate the kidney’s fibrosis level. The results showed that elevated TGF-β1, α-SMA, and MMP2 expression was significantly reduced after the coadministration of SCG. The elevated TGF-β1 and MMP2 were also significantly decreased after the coadministration of VAL. Interestingly, Shaun AS et al. also found a significant reduction in elevated renal TGF-β1 and α-SMA expression with the coadministration of SCG in the progression of unilateral ureteral obstruction (UUO)-induced renal fibrosis [26]. It is well known that activating the TGF-β1/Smad2/3 pathway is an essential pathogenic mechanism leading to tissue fibrosis [18]. Activation of TGF-β1 triggers nuclear localization of Smad2/3 along with overexpression of the fibrosis-promoting gene [17]. Therefore, the Smad2/3 pathway was also examined, and it was found that the coadministration of SCG reduced the phosphorylation level of Smad2/3. These results suggest that the combination of SCG alleviates renal fibrosis by inhibiting TGF-β1/Smad2/3 pathways.

Oxidative stress is one of the main causes of renal fibrosis. At the same time, renal dysfunction often triggers oxidative stress and accelerates kidney disease, such as acute kidney injury (AKI), CKD, and nephrotic syndrome [52]. Persistent hyperuricemia can lead to various disorders, including hypertension, renal vasoconstriction, tubular damage, renal oxidative stress, mitochondrial dysfunction, and reduced ATP levels [53]. UA acts as the ultimate product of purine degradation in humans. Although UA is reportedly present in plasma as an antioxidant, it behaves as a powerful pro-oxidant once high concentrations of UA enter the cells [54]. The mechanism of the body’s defense against oxidative damage operates in two principal systems, namely the scavenging of free radicals and reactive substances through enzymes (e.g., GSH-Px, SOD) and electron donors (e.g., GSH, tocopherols, ascorbic acid) [55]. The results of renal oxidative stress were also evaluated, and it was found that it reduced levels of GSH, GSH-Px, and GR, which were elevated by the coadministration of SCG or VAL. In the kidney, Ang II leads to excessive production of reactive oxygen species (ROS), renal vasoconstriction, and the release of pro-inflammatory mediators (IL-6 and TGF-β) through the activation of NADPH oxidase (NOX), ultimately leading to renal fibrosis [56]. Ang II also triggers the overproduction of mitochondrial superoxide and mitochondrial structural and functional dysfunction [57]. Furthermore, Ang II has been reported to aggravate liver fibrosis by inducing NOX-dependent oxidative stress [58]. Ang II-induced erythrocyte senescence partially due to enhanced oxidative stress [59]. In sepsis-associated thrombocytopenia, elevated Ang II directly stimulates platelet apoptosis in an angiotensin II type 1 receptor (AT1R)-dependent manner by promoting oxidative stress [60]. Moreover, long-term Ang II administration for 28 days was found to cause cardiac insufficiency, hypertrophy, fibrosis, apoptosis, and oxidative stress in mice [61]. In contrast, Wakayama et al. found that vaccination with Ang II successfully produced anti-Ang II antibodies in serum and had neuroprotective and antioxidant effects against cerebral ischemia [62]. In vitro, Sanchez-Calvo found that treating human kidney-2 (HK-2) cells with Ang II increased superoxide, nitric oxide, and hydrogen peroxide levels, induced nitric oxide synthase expression, and produced mitochondrial dysfunction [63]. Milanesi et al. found that Ang II promotes inflammation and oxidative stress in HK-2 cells by activating Toll-like receptor 4 (TLR 4) [64]. Wang et al. used the Ang II treatment of HK-2 to induce epithelial-to-mesenchymal transition (EMT) [65]. The mechanism involved may be related to the negative regulatory effect of Ang II on the expression of sirtuin 3 (SIRT3) and peroxidase [66]. It is well known that renin is a crucial rate-limiting enzyme in the renin–angiotensin system (RAS), and Veerappan et al. showed that renin release from MCs promoted Ang II production, triggered airway RAS and promoted bronchoconstriction [67]. The serum Elisa testing found elevated Ang II levels, which were significantly reduced after the coadministration of SCG or VAL.

Furthermore, in vitro experiments with stimulation of RBL-2H3 cells by UA also revealed a significant increase in the release of Ang II from RBL-2H3 cells after 24 or 48 h incubation with UA. Considering that Ang II causes an increase in renal oxidative stress levels, leading to renal fibrosis, the promotion of Ang II production by MCs is also a non-negligible trigger for the development of renal fibrosis. The kidneys require many mitochondria to remove waste products produced by the body and maintain the balance of the body’s internal environment [36]. The kidney has one of the highest mitochondrial densities [33]. It is one of the most energy-intensive organs in the body, second only to the heart in terms of mitochondrial content and energy expenditure [68]. We also found significant alleviation of mitochondrial structural damage in response to the coadministration of SCG or VAL. These results suggest that the coadministration of SCG or VAL may attenuate renal damage by depressing Ang II production, alleviating mitochondrial structural damage, and decreasing renal oxidative stress.

## 4. Materials and Methods

### 4.1. Chemicals and Reagents

Solarbio Biotechnology (Beijing, China) supplied adenine. Aladdin Biotechnology (Shanghai, China) provided sodium cromolyn (SCG) and valsartan (VAL). Nanjing Jiancheng Bioengineering Institute (Nanjing, China) provided kits for the detection of renal glutathione (GSH), glutathione peroxidase (GSH-Px), glutathione reductase (GR), and malondialdehyde (MDA) levels. MILIAN Biotechnology (Shanghai, China) offered an ELISA kit for the detection of Angiotensin II (Ang II) levels. OXOID Ltd. (Basingstoke, UK) provided yeast extract. Thermo Fisher Scientific (Waltham, MA, USA) provided RIPA buffer. Solarbio Biotechnology (Beijing, China) supplied SDS-PAGE loading buffer and phenylmethylsulfonyl fluoride (PMSF). Solarbio Biotechnology (Beijing, China) supplied the reactive oxygen species assay kit. MedChemExpress (Monmouth Junction, NJ, USA) supplied Angiotensin II human. Cell Bank of Type Culture Collection of the Chinese Academy of Sciences provided the RBL-2H3 and HK-2 cell line. Hyclone (Auckland, NZ, USA) provided MEM medium, trypsin, and antibiotic-antimycin. Gibco (Grand Island, NY, USA) supplied fetal bovine serum (FBS). In addition, Sigma-Aldrich (St. Louis, MO, USA) supplied all other chemical reagents.

### 4.2. Animals

Eight-week-old male Sprague-Dawley (SD) rats weighing between 180 and 220 g were provided by the Lanzhou University Animal Experiment Center (production license number: No. SCXK (Gan) 2020-0002). All animals were housed in the SPF animal house at Lanzhou University and maintained at conditions of 24 °C temperature, 40% humidity, and a 12-h light/dark cycle. Animal experiments were carried out under the guidelines of the Animal Ethics Committee of the First Affiliated Hospital of Lanzhou University (ethics approval number: LDYYLL 2019-141). During the experiment, all rats drank sterilized pure water and food freely. In addition, the experimental procedures and animal care were carried out in accordance with the principle of minimizing animal suffering. Animals were used for experimental studies after one week of acclimatization. The experimental scheme is shown in Table 1. Rats in the hyperuricemia group were given adenine 100 mg·kg^−1^·d^−1^ by intragastric administration and fed with 10% yeast powder for three consecutive weeks. Rats in the control group were fed normal food and pure water. In the fourth week, the rats in the hyperuricemia group were redivided into three groups according to their body weight: the model group, the sodium cromoglycate (SCG) group, and the valsartan (VAL) group, with seven rats in each group. Rats in the SCG and VAL groups were respectively given 25 mg·kg^−1^ SCG intraperitoneally and 50 mg·kg^−1^ VAL by gavage. Rats in the model and control groups were given equal volumes of saline intraperitoneally and pure water by gavage once daily for three weeks. The body weight was recorded every two days.

### 4.3. Biochemical Analysis

Following a 24-h fasting period after the last treatment, approximately 3 mL blood samples were collected from the abdominal aorta under anesthesia. Blood samples were centrifuged at 8000 rpm for 10 min to collect the serum. The automatic biochemical analyzer (OLYMPUS AU400, Tokyo, Japan) was used to measure the levels of blood urea nitrogen (BUN), creatinine, uric acid (UA), and cystatin C, which serve as indicators of renal function. The remaining serum samples were stored in the −80 °C freezer for subsequent testing.

### 4.4. Observation of MCs 

After the blood samples were collected, the kidneys were collected with surgical scissors, rinsed with normal saline, and dried with absorbent paper. The total kidney weight was recorded, and the left kidney was placed in liquid nitrogen and transferred to a −80 °C freezer. Part of the tissues of the right kidney was collected and fixed in 10% formaldehyde at room temperature for 24 h. Part of the kidney tissue was cut and placed in a pathological embedding box. The excess formaldehyde on the tissue surface was removed with running water. The tissue was dehydrated, embedded according to conventional procedures, and prepared into 3 µm tissue slices. Twenty-five non-overlapping areas were randomly selected to quantify MCs through observation and photography under an optical microscope.

### 4.5. Western Blotting Analysis

The cryopreserved kidney samples were chopped with surgical scissors and put into a centrifuge tube. RIPA buffer was added to dissolve the tissue and then centrifuged at 14,000 rpm for 10 min to collect the supernatant. A BCA kit was used to measure the protein concentration. After withdrawing an appropriate amount of supernatant, SDS-PAGE loading buffer was added with mixing and boiled for 5 min to denature the protein. Proteins with different molecular weights were separated by SDS-PAGE electrophoresis and transferred to methanol-activated PVDF membranes. The PVDF membrane was sealed with 5% skim milk (dissolved in TBST) for one h and then incubated with anti-SCF (sc13126, Santa Cruz Bio., Dallas, TX, USA), anti-c-kit (sc365504, Santa Cruz Bio., Dallas, TX, USA), anti-TGF-β1 (sc130348, Santa Cruz Bio., Dallas, TX, USA), anti-α-SMA (GB111364, Servicebio Bio, Wuhan, China), anti-MMP2 (ab181286, Abcam plc, Cambridge, UK), anti-MMP9 (ab76003, Abcam plc, Cambridge, UK), anti-Smad2/3 (sc133098, Santa Cruz Bio., Dallas, TX, USA), anti-p-Smad2/3 (abs130992, Absin Bio, Shanghai, China), and anti-β-actin (4970s, Cell Signaling Technology, Danvers, MA, USA) antibody at 4 °C overnight. The membranes were then incubated with HRP-conjugated secondary antibody for 1 h, and TBST was used for background reduction. The signals were detected using an enhanced chemiluminescence (ECL) solution and developed with an Automatic Chemiluminescence/Fluorescence Image Analysis System (Tanon 4600 Series, Shanghai, China). The gray values were statistically analyzed using Image J software (National Institutes of Health, Bethesda, MD, USA).

### 4.6. Histopathological Evaluation

HE and Masson staining were performed according to the instructions of the staining kit from Servicebio Biotechnology Co., Ltd. (Wuhan, China). Kidney sections were dewaxed to water, subjected to hematoxylin and eosin staining for HE staining, and then dehydrated and sealed. Similarly, kidney sections were dewaxed to water for Masson staining and subjected to overnight potassium dichromate and subsequent staining with iron hematoxylin, phosphomolybdic acid, and aniline blue. The sections were then dehydrated, sealed, and observed under a microscope.

### 4.7. Observation of Mitochondrial Structure 

Part of the kidney tissues was transferred to an electron microscope fixation solution containing 2.5% glutaraldehyde at 4 °C. The kidney tissue was removed from the fixative, rinsed with phosphate buffer, fixed with 1% osmium tetroxide solution for 1 h, and then gradient-dehydrated in ethanol. We embedded samples in resin and cut ultrathin sections with a glass knife using an ultramicrotome (EM UC7/FC7, Leica Microsystems, Wetzlar, Germany). The ultrathin slices were placed on carbon-coated copper grids and examined with a field emission high-resolution transmission electron microscope (HRTEM, Tecnai G2 F30, FEI Company, Hillsborough, Oregon, USA). Photographic images were captured using a CCD camera (Gatan 894 Ultrascan 1000 camera, Gatan, Pleasanton, California, USA).

### 4.8. Oxidative Stress Evaluation

To assess renal oxidative stress levels, glutathione (GSH), glutathione peroxidase (GSH-Px), glutathione reductase (GR), and malondialdehyde (MDA) levels were measured. A portion of the kidney sample was weighed, washed with normal saline, and dried with absorbent paper. The tissue was then homogenized, and the homogenate was centrifuged at 5000 rpm for 10 min to collect the supernatant for testing. The GSH, GSH-Px, and MDA levels were determined at 405 nm, 412 nm, and 532 nm, respectively, following the manufacturer’s instructions (Nanjing Jiancheng Bioengineering Institute, Nanjing, China). Distilled water was used to adjust the baseline to zero.

### 4.9. Cell Culture and Cell Viability Assay

MEM medium with 10% fetal bovine serum (FBS) and 1% antibiotic-antimycin was used to culture RBL-2H3 or HK-2 cells, and the incubator was set at 37 °C and 5% CO_2_. Cell Counting Kit-8 (CCK-8) (Beyotime, Haimen, China) was used to assess cell viability, and the Moxi-Z automatic cell counter (Orlfo Technologies, Ketchum, ID, USA) was used to quantify the cultured cells. RBL-2H3 or HK-2 cells were cultured in a 96-well plate at the concentration of 5 × 10^6^/mL for 24 h. The cells were incubated with fresh, serum-free medium containing different concentrations of uric acid or Ang II for 24 and 48 h, respectively. After cultured at 37 °C for 2 h, CCK-8 reagent was added for coloration. The absorption coefficient of each hole was measured at 450 nm wavelength, and its activity was determined by measuring the ratio of the absorption coefficient of the hole treated with uric acid to that of the control group. 

### 4.10. Detection of Ang II Release from Rat Serum and RBL-2H3 Cells

The frozen rat serum samples were defrosted on ice. The culture medium of RBL-2H3 cells was collected for 24 or 48 h, and protein concentration was measured using a BCA kit. Standard and sample wells were set up according to the instructions of the ELISA kit (MILIAN, China). Standard wells were spiked with 50 μL of different concentrations of the standard, and sample wells received 40 μL of sample dilution followed by the addition of 10 μL of the sample. Then, 100 μL of the test solution containing HRP was added to each well and incubated at 37 °C for 60 min. The wells were washed, incubated for 30 s, and drained five times. Color development reagent (100 uL) was added to each well and incubated for 15 min at 37 °C while being protected from light. Afterward, 50 uL of termination reagent was added to each well to terminate the color development reaction, and the absorbance of each well at 450 nm was determined.

### 4.11. Detection of Reactive Oxygen Species

HK-2 cells were cultured in 20 mm glass-bottomed dish at the concentration of 1 × 10^5^/mL for 24 h. The cells were incubated with fresh, serum-free medium containing different concentrations of Ang II for 24 and 48 h. DCFH-DA was first diluted with serum-free MEM medium to a concentration of 10 μM. The cell medium was then removed, and the appropriate diluted DCFH-DA-containing medium was added and incubated for 20 min at 37 °C in a cell incubator. The cells were washed three times with serum-free MEM medium to remove any DCFH-DA that had not entered the cells fully. Detection of reactive oxygen species using fluorescence microscopy (488 nm excitation wavelength, 525 nm emission wavelength).

### 4.12. Statistical Data Analysis

The data were processed by IBM SPSS 17.0 software. Independent sample *t*-test and one-factor ANOVA were carried out for the obtained data. The results were calculated by mean ± standard deviation, and there was a significant difference (*p* < 0.05).

## 5. Conclusions

In summary, renal fibrosis caused by hyperuricemia may be closely related to MC recruitment. The activation of SCF/c-kit leads to MC activation, promoting renal fibrosis through the TGF-β1/Smad2/3 pathway and aggravating renal oxidative stress injury. MC stabilizers such as SCG may alleviate hyperuricemia-induced renal fibrosis by inhibiting the recruitment of MCs and suppressing the TGF-β1/Smad2/3 pathway, and attenuate mitochondrial structural damage and relieve renal oxidative stress by inhibiting the production of Ang II.

## Figures and Tables

**Table 1:** Groups and treatment.

	Protocol One			Protocol Two	
Groups	Treatment (daily for three weeks)		Treatment (daily for three weeks)		
	Food	Oral gavage (o.g.)	Groups	Intraperitoneal injection (i.p.)	Oral gavage (o.g.)
Control	Normal food	1% gum acacia solution (1 mL/100 g)	Control	saline (0.25 mL/100 g)	saline (0.25 mL/100 g)
Model	High purine food (contained 10% yeast powder)	Adenine (100 mg/kg)	Model SCG	saline (0.25 mL/100 g) SCG (25 mg/kg)	saline (0.25 mL/100 g) saline (0.25 mL/100 g)
			VAL	saline (0.25 mL/100 g)	VAL (50 mg/kg)

## Data Availability

The data generated by this study will be made available upon request.
